# Schistosomiasis in migrant children and adolescents in a paediatric tropical referral unit in Spain: diagnosis and long-term management challenges

**DOI:** 10.1007/s00431-024-05623-2

**Published:** 2024-08-15

**Authors:** Paula Rodríguez-Molino, Soraya González Martínez, Jorge Bustamante Amador, Isabel Mellado-Sola, Laura Montes Martín, Iker Falces-Romero, Milagros García López-Hortelano, Jara Hurtado-Gallego, María José Mellado, Carlos Grasa, Talía Sainz

**Affiliations:** 1 General Paediatrics, Infectious and Tropical Diseases Department, Hospital La Paz, Madrid, Spain; 2 La Paz Hospital Research Institute (IdiPAZ), Madrid, Spain; 3 Autonomous University of Madrid (UAM), Madrid, Spain; 4 Centro de Investigacion en Red en Enfermedades Infecciosas (CIBERINFEC), Madrid, Spain; 5 Health Centre Alameda de Osuna, Madrid, Spain; 6 Hospital Paediatrics Department, Childhood Infections, El Escorial Hospital, Madrid, Spain; 7 Sanitas la Moraleja University Hospital, Madrid, Spain; 8 Department of Microbiology and Parasitology, La Paz University Hospital, Madrid, Spain; 9 Translational Research Network in Paediatric Infectious Diseases (RITIP), Madrid, Spain; 10 Health Centre Guzmán el Bueno, SERMAS, Madrid, Spain

**Keywords:** Schistosomiasis, Migrants, Children and adolescents, Screening, Parasites

## Abstract

Globalisation and population movement have led to an increasing number of migrant children residing in areas non-endemic for schistosomiasis. However, diagnosing and managing schistosomiasis in children remain controversial. This study aims to investigate the prevalence of schistosomiasis in migrant children and to describe the diagnostic approach and management strategies, including long-term follow-up, to explore the potential role of serological tests in evaluating treatment response. We conducted a retrospective descriptive study spanning from January 2014–July 2021 at a referral unit for Paediatric Tropical Diseases in Madrid (Spain). The study included patients under 18 years diagnosed with schistosomiasis. Of 679 children screened for schistosomiasis, 73 (10.8%) tested positive. The median age was 16.3 years [IQR 9–17.6], 74% male. The majority originated from Sub-Saharan Africa (47%) and Asia (47%). Only 40% presented with symptoms, with gastrointestinal (18%) and cutaneous (17%) manifestations being the most common. Eosinophilia was observed in 43% (median [IQR]: 1103/mm3 [671–1536]), and ova were visualised in the urine of 2/50 (4.0%). Praziquantel treatment was administered to 92%, and 5 patients required retreatment. Follow-up data were available for 58 (80%) over a median period of 9 months [IQR 6–19.8], revealing a progressive decline in eosinophil count, IgE titres, and ELISA optical density.

*    Conclusion*: In this series, the prevalence of schistosomiasis among migrant children was significant (10%), highlighting the importance of including serological tests in migrant health screening. The disease is largely asymptomatic, eosinophilia is often absent, and visualisation of ova in urine is exceedingly rare. Eosinophil count, IgE titres, and ELISA optical density could prove valuable as an initial approach for monitoring inflammation during follow-up assessments.

**Table:** 

**What is Known:** *• The burden of disease related to schistosomiasis is significant, particulary in children, and it is advisable to screen this vulnerable population.*	
**What is New:** *• Eosinophilia may not be present in parasitic infections, so serological tests are crucial for screening migrant children.* *• Serological monitoring facilitates long-term management of migrant children with schistosomiasis.*

**What is Known:**

*• The burden of disease related to schistosomiasis is significant, particulary in children, and it is advisable to screen this vulnerable population.*

**What is New:**

*• Eosinophilia may not be present in parasitic infections, so serological tests are crucial for screening migrant children.*

*• Serological monitoring facilitates long-term management of migrant children with schistosomiasis.*

## Introduction

Schistosomiasis, also known as bilharziasis, is a parasitosis included among the neglected tropical diseases by the World Health Organization (WHO) [1]. The disease, caused by a blood trematode for which humans are the definitive host, is acquired through contact with contaminated freshwater via skin penetration. The primary species affecting humans—*Schistosoma haematobium, S. japonicum, and S. mansoni*—are predominantly distributed across Africa, the Middle East, Asia, certain regions of South America, and the Caribbean, often linked to limited access to safe water and inadequate sanitation [2].

Although schistosomiasis is recognised for inducing Katayama fever, a substantial number of cases remain asymptomatic. Untreated, even a low parasite burden can trigger a pro-inflammatory response, leading to chronic morbidities such as urinary tract fibrosis and hepatosplenic disease [2]. Long-term consequences are abdominal pain, hepatomegaly, haematuria, and urogenital complications, including cancer and infertility, as well as spinal cord compression [3, 4]. Complications afflict 4%–10% of adults at the time of diagnosis [5], rendering the burden of schistosomiasis economically and medically substantial, given that the disease incapacitates more individuals than it claims.

Due to behavioural and hygiene patterns, school-aged children are particularly susceptible to schistosomiasis. In endemic areas, many children acquire the infection by the age of 2 years and live with chronic infection [6]. Early-life complications, stemming from damage to epithelial barriers, manifest as anaemia, poor nutrition, and growth impairments [1]. Since 2006, the WHO has embraced a mass treatment strategy, with over 236 million people treated in endemic countries in 2019 [6]. Nonetheless, the cure rate notably decreases in younger children and those with history of previous treatment [7].

Diagnosis and management strategies in non-endemic areas remain contentious, with numbers escalating, primarily due to population movements. In Europe, sporadic outbreaks due to local transmission can occur, and human cercarial dermatitis is frequently observed, primarily due to the presence of avian schistosomiasis in several countries across the continent [8]. In a recent series in non-endemic areas, 19% of migrant children were diagnosed with schistosomiasis [9]. Microscopy techniques, while highly specific, have limited sensitivity for low-intensity infections and post-treatment monitoring due to variability in egg excretion within individuals. Emerging antigen tests, like the Up-Converting Reporter Particle Technology-based Lateral Flow (UCP-LF) Circulating Cathodic Antigen (CAA) test, show promise with high specificity and sensitivity for detecting all human *Schistosoma* species, but commercial availability is pending [10].

Therefore, screening asymptomatic migrant patients primarily relies on serology as the first-line test, lacking specificity and being prone to cross-reactivity with other helminth infections due to shared antigenic determinants [10]. These tests can persist as positive and do not discriminate against acute infection [3, 11] or distinguish between *Schistosoma* species [12, 13]. In travellers to endemic areas and in children, antibody detection appears more sensitive than parasitological methods [11, 14]; however, data regarding long-term serological evolution are sparse.

This study aims to delineate the prevalence of schistosomiasis in a cohort of children and adolescents screened for imported diseases at a Paediatric Tropical Reference Unit in Spain. We delineate risk factors, diagnostic protocols, and management strategies, including long-term follow-up, to explore the potential role of serology in evaluating treatment response. Optimising the management of paediatric migrant populations is pivotal in defining evidence-based protocols for screening imported diseases.

## Materials and methods

### Study design and population

A retrospective, observational study was conducted at a National Reference Unit for Tropical Infectious Diseases and International Adoption in Madrid, Spain, attending patients referred from primary care, general paediatrics, and reception centres for asylum seekers and refugees, for the screening of imported diseases. The study included all patients younger than 18 years of age diagnosed with schistosomiasis through serological tests and/or visualisation of eggs in urine or faeces between January 2014 and July 2021. Patients were selected through microbiology and electronic medical records from the unit. Those with missing relevant data (i.e. sex, origin, symptoms, co-parasitisations) or lost to follow-up were excluded.

All patients underwent standardised management according to a uniform protocol [9], encompassing haemogram and biochemistry, immunoglobulins, and faecal and urine tests. Serum and 3 faecal samples, which were collected on alternate days, were sent to the microbiology laboratory. Serological test for *Strongyloides stercoralis* and *Toxocara* spp. were performed for all patients as part of the health screening protocol. Serological tests for schistosomiasis were conducted only for children arriving from endemic areas. A mid-morning urine collection after exercise was performed in patients with altered urine analysis and positive serology, for the study of eggs.

All patients were managed based on clinician-defined criteria. Epidemiological and clinical data, including clinical presentation, microbiological results, coinfections, and treatment-related information were electronically collected at the first medical visit and stored in an anonymised database. Follow-up data included symptoms, physical examinations, blood tests, and microbiological workup, including repeated serologies, until discharge.

Eosinophilia was defined by a threshold of 500 eosinophils/microL [15] and anaemia was defined based on age [16]. Elevated immunoglobulin (Ig) E levels were considered when > 100 U/mL [15] whereas thresholds for IgG, IgM, and IgA varied with age. Regarding liver function, hypertransaminasaemia was considered when AST was > 50 U/L or ALT > 45 U/L [17].

The study was conducted in accordance with the Declaration of Helsinki and received ethical approval from the Hospital's Ethics Committee (PI-3348). No informed consent was required due to the retrospective design of the study.

### Parasitological diagnosis

Both direct and indirect methods were employed for the diagnosis of *Schistosoma* infection. Direct techniques involved microscopic detection of *Schistosoma* ova in stool or urine samples after filtration. Millipore Swinnex^®^ membrane filter holders (25 mm) and Whatman Nuclepore^®^ polycarbonate membranes (10 μm) were utilised, with a minimum of 150 mL of urine required for filtration.

### Serological diagnosis

Indirect diagnostic tools included a commercial serologic test for *Schistosoma*, employing an enzyme-linked immunosorbent assay (ELISA) against SmSEA antigen (IgG-ELISA NovaTec Immunodiagnostica GmbH^®^, Germany). A reactive result was defined by an optical density value > 1.1; indeterminate from 0.9–1.1; and non-reactive when < 0.9, following the manufacturer’s instructions.

Concurrently, within our screening protocol, commercial serological kits for Strongyloides (SciMedx microwell ELISA^®^) and Toxocara (NovaTec Immundiagnostica GmbH^®^, Germany) were employed. Diagnoses for strongyloidiasis and toxocariasis were made based on serological test results.

### Statistical approach

Continuous variables were described using medians and their interquartile ranges (IQRs), whereas categorical variables were described using absolute and relative frequencies. Longitudinal data were analysed at various time points according to the visits and represented using Prism 9.0. All analyses were performed with IBM SPSS Statistics version 23 and/or Prism 9.0.

## Results

### Study participants

Of the 679 children tested for schistosomiasis during the study period, 77 exhibited a positive serological result for *Schistosoma* spp. Four patients were excluded, 1 due to the absence of a compatible epidemiological history, and 3 due to discordant serology results without treatment (serology tested negative before starting treatment). The estimated overall prevalence in our cohort was 10.8%.

**Fig. 1:**
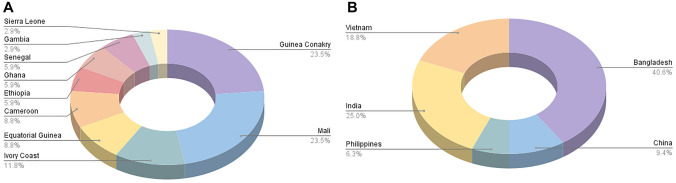
Country of origin of patients from Sub-Saharan Africa (**A**) and from Asia **(B**)

The majority of cases were in males originating from Sub-Saharan Africa and Asia, with a median age of 16.3 years [IQR 9–17.6], the majority (52/73, 71%) being above 10 years old. The country of origin of patients from Sub-Saharan Africa is shown in Fig. 1A, and from Asia in Fig. 1B. A significant proportion (60%) of patients were asymptomatic, and eosinophilia was present in only 43% of cases, with mild elevation observed in most instances (median eosinophil count among those with eosinophilia: 1103 [671–1536]). The majority of patients with symptoms (76%) were over 10 years old. *Schistosoma* spp. ova from urine sample were visualised in only 2 (4.0%) of 50 patients, both of whom presented with haematuria. Detailed demographic data are summarised in Table 1, and clinical, laboratory, and microbiological data are presented in Table 2.

**Table 1:** Epidemiological data of the patients included in the study

Total patients *N* = 73	
**Age** (years)^a^	16.3 [9–17.6]
**Sex** (*N*, %)	
Male	54 (74%)
Female	19 (26%)
**Condition in Spain** (*N*, %)	
Immigrant	27 (38%)
Adoptee	21 (29%)
Refugee	20 (28%)
VFR	3 (4.1%)
Spanish traveller	1 (1.4%)
**Origin**	
Sub-Saharan Africa	34 (47%)
Asia	32 (44%)
South America	2 (2.7%)
Central America	2 (2.7%)
Maghreb	2 (2.7%)
Europe	1 (1.4%)
**Time after exposure** (days)^a^	90 [30–120]
**Freshwater baths**	23 (32%)
**Reason for consultation** (*N*, %)	
Health control after adoption	21 (29%)
Eosinophilia	12 (16%)
Screening of tropical pathology	23 (32%)
Tuberculosis screening	17 (23%)
**Medical background**	30 (41%)
Previous parasitisations	13 (18%)
* Plasmodium*	9 (12%)
* Giardia*	2 (2.7%)
* Strongyloides*	1 (1.4%)
* Toxocara*	1 (1.4%)
* E*. *vermicularis*	1 (1.4%)
Tuberculosis	5 (6.8%)
HBV infection	3 (4.1%)
HCV infection	2 (2.7%)
HIV infection	1 (1.4%)

*VFR* visiting friends and relatives, *HBV* hepatitis B virus, *HCV* hepatitis C virus, *HIV* human immunodeficiency virus

^a^median value [*IQR* interquartile range]

**Table 2:** Clinical laboratory microbiological results of the study participants

**Clinical manifestations**	29 (40%)
Cutaneous	11 (15%)
Skin pruritus	8 (11%)
Anal pruritus	3 (4.1%)
Gastrointestinal	13 (18%)
Persistent diarrhoea	8 (11%)
Abdominal pain	3 (4.1%)
Vomiting	1 (1.4%)
Haematuria	4 (5.5%)
**Physical examination**	
Eczema	4 (5.5%)
Abdominal swelling	3 (4.1%)
Abdominal pain	1 (1.4%)
Visceromegaly	1 (1.4%)
**Somatometry**^b^	
BMI^a^	20 [15.4–22.3]
Low weight for age	11 (16%)
Low height for age	19 (28%)
Low BMI for age	8 (12%)
**Analytical results**	
Anaemia	16 (22%)
Eosinophilia	31 (43%)
< 1000/microL	12 (17%)
1000–1500/microL	10 (14%)
> 1500/microL	9 (13%)
Leukocytosis	6 (8.2%)
Increased IgE	23/31 (74%)
IgE (KU/L)^a^	889 [177–2369]
Hypertransaminasaemia	8 (11%)
**Positive serology for *Schistosoma* spp.**	73 (100%)
OD ELISA^a^	2.7 [1.7–4.5]
***Schistosoma haematobium* ova in urine sample**	2/48 (4.2%)
**Co-parasitisations**	44 (60%)
**Positive serology for other helminths**	19/45 (42%)
* Strongyloides stercoralis*	10/45 (22%)
* Toxocara canis*	11/43 (26%)
**Other positive parasites**	35/68 (52%)
* Ascaris*	3/68 (4.4%)
* Oxiuros*	1/68 (1.5%)
* Anisakis*	1/68 (1.5%)
* Taenia saginata*	1/68 (1.5%)
* Giardia*	6/68 (8.8%)
Non pathogenic protozoa	24/68 (35%)
* Blastocystis* spp.	15/68 (22%)
* Entamoeba coli*	2/68 (2.9%)
* Endolimax nana*	6/68 (8.8%)
**Abdominal ultrasounds**	25 (34%)
Hepatomegaly	3/25 (12%)
Liver hypoechogenicity	1/25 (4.0%)
Portal hypertension	1/25 (4.0%)
Uropathy	1/25 (4.0%)
Total patients *N* = 73	

Variables: *N*/*N* (%): Number of analysed patients/Number of total patients (%) (Number of total patients is 73, unless stated otherwise)

*VFR* visiting friends and relatives, *HBV* hepatitis B virus, *HCV* hepatitis C virus, *HIV* human immunodeficiency virus, *BMI* body mass index, *IgE* immunoglobulin E, *OD* optical density value, *ELISA* enzyme-linked immunosorbent assay

^a^median value [IQR (interquartile range)]

^b^Compared with the WHO child growth standards

### Treatment

Of the 73 patients, 67 underwent treatment with praziquantel (median dose 40 mg/kg/day [40–58], administered 2 or 3 times a day, for 1–3 days). Six patients did not receive treatment because of loss to follow-up (4 cases) or treatment deferral due to hepatitis B virus and latent tuberculosis infection, leading to subsequent loss to follow-up. Praziquantel was generally well-tolerated, and no adverse events were reported, except for 1 patient previously diagnosed with autoimmune hepatitis and hyperreactive malarial splenomegaly syndrome who exhibited hypertransaminasaemia after the first dose. Treatment was discontinued and successfully completed before liver transplantation.

In cases of co-parasitisation requiring multiple drugs, the treatment order was determined by the managing clinician. Seven (7/23) patients were co-parasitised with *Toxocara* spp.: 6 received albendazole and praziquantel; and 1, in whom *Toxocara* spp. serology decreased over follow-up, only received praziquantel. Co-parasitisation by *Strongyloides stercoralis* was present in 5 patients, and all received ivermectin. Two patients co-parasitised by *Toxocara* spp. and *Ascaris* (2/23) first received praziquantel and secondly mebendazole. Only 1 patient was co-parasitised by *Toxocara* spp. and *Strongyloides*, receiving praziquantel followed by albendazole with a decrease in *Strongyloides* serology after toxocariasis treatment, and therefore was never treated for strongyloidiasis. *Giardia lamblia* was present in only 1 patient, treated first with metronidazole to ensure absorption of praziquantel. One patient presented with ascariasis and received mebendazole followed by praziquantel.

### Follow-up

Follow-up data were available for 58 (80%) of 73 patients, including 1 patient who did not receive praziquantel, with a median follow-up time of 9 [IQR 6–19.8] months. The initial follow-up visit occurred at a median of 4 [IQR 1.4–6] months. Amongst the 28 symptomatic patients at the first evaluation, 13 (46%) reported persistent symptoms, and 18 (64%) still exhibited eosinophilia, with a median eosinophil count among those with persistent eosinophilia of 1045/microL [IQR 938–1270]. Overall, in 27 of 48 patients, the total eosinophil count decreased over the first months (delta = −343 [−160 to −967]). Serological tests for *Schistosoma* in the initial follow-up visit turned negative in 5 (8.6%) patients, were indeterminate in 4 (7.3%), and remained positive in 46 (84%) of 58.

**Fig. 2:**
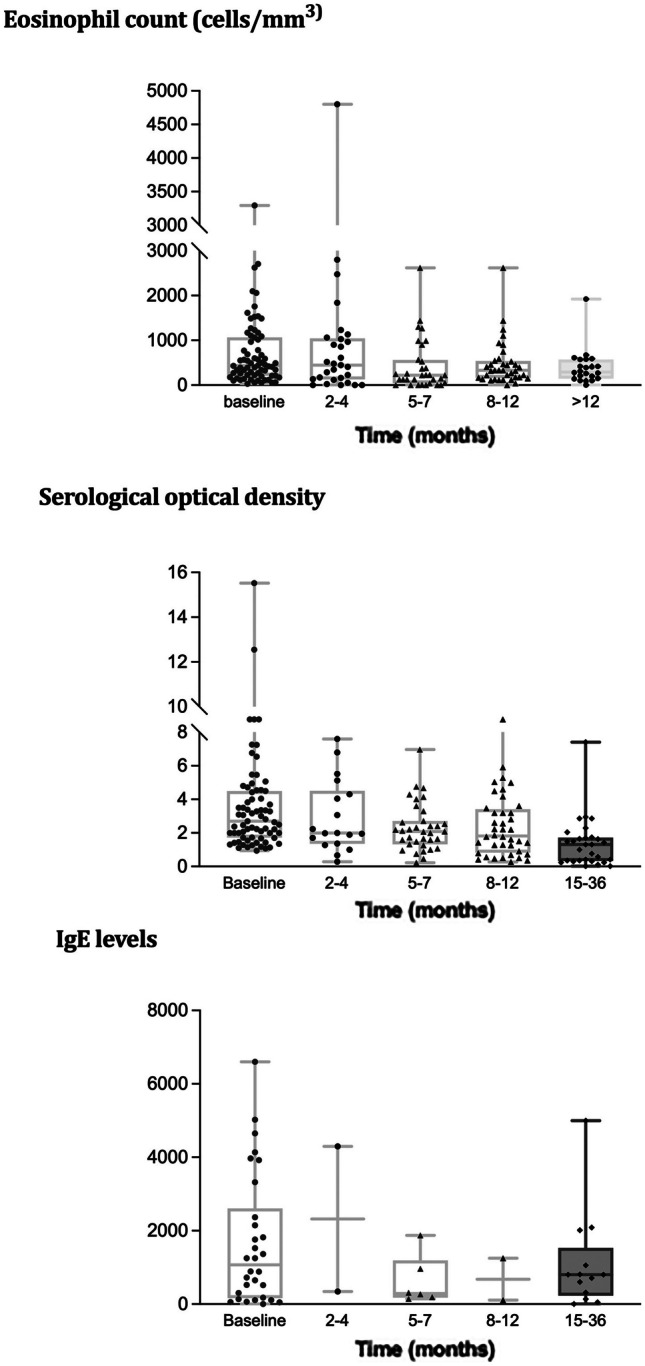
Peripheral eosinophils, serum IgE. and ELISA optical density over follow-up. Legend: IgE (KU/L): immunoglobulin E; OD: optical density

Only 51 patients had long-term follow-up, and over time, serology became negative in only 15 (26%) patients, with a median time to sero-reversion of 9 [IQR 4–16.9] months. However, a progressive decline in ELISA optical density, total IgE, and peripheral eosinophil count was observed over time. The evolution of these parameters is illustrated in Fig. 2.

Five (7.5%) patients underwent retreatment during follow-up. The reasons for retreatment included persistent haematuria and/or priapism (2 patients), persistently positive serology (2 patients), and/or ova visualisation in urine despite treatment (1 patient). Three patients received 2 cycles of praziquantel, and 2 received 3 cycles of treatment, without experiencing relevant adverse effects.

## Discussion

In this cohort of migrant and internationally adopted children arriving in Europe attending a Paediatric Tropical Referral Unit for health screening, we found a 10% seroprevalence of schistosomiasis among those arriving from endemic areas. The majority of patients were asymptomatic, and less than half of them presented with eosinophilia. The detection of ova in urine was exceedingly rare and was associated with haematuria. Notably, our series did not observe significant complications, possibly due to the young age of the patients, which contrasts with findings in other series [18]. All patients underwent treatment with praziquantel, which was generally well-tolerated, with only 5 patients requiring retreatment. Longitudinal follow-up revealed a decline in absolute eosinophil counts, ELISA optical density, and total IgE post-treatment.

Despite the majority of patients originating from sub-Saharan Africa and predominantly presenting with gastrointestinal symptoms, no *Schistosoma mansoni* or *intercalatum* was detected in stool. In studies conducted in high-burden settings, *S. mansoni* was detected in faeces in 74% of screened children [19], but conventional microscopy is designed to identify moderate-to-high intensity infections [10]; however, data from non-endemic countries remain scarce.

Series addressing schistosomiasis in children are limited, with a previous series in our country focusing on adult patients reporting a prevalence of 32% among African migrants [20]. However, the prevalence is known to increase gradually during childhood up to adolescence/early adulthood, estimating a burden of disease among schoolchildren from endemic areas of approximately 60%–80% [2]. The observed difference in prevalence in our study, with a median age of 16 years, might be attributed to age-related variations, given that adults more frequently present with symptoms and have a higher risk of serious complications [21].

Studies on travelling children from Asia and Africa, where most migrant minors to Europe also come from [22], report high infection rates, reaching up to 90% among those exposed, with lower eosinophil counts compared with the adult population [23]. However, other groups addressing screening strategies amongst paediatric refugees in the US found a prevalence of eosinophilia by place of origin ranging from 17%–20%, with a positive predictive value for identifying positive *Schistosoma* serology of 0.23% [24]. These findings suggest that absolute eosinophil count might not be a reliable screening tool in migrant children.

Diagnosing schistosomiasis in children is challenging due to the often absent symptoms, infrequent abnormal blood tests or ultrasounds, and lower sensitivity of parasitological methods in the paediatric population [11], unlike what happens with other common parasites in children, such as *Blastocystis sp*. or *G. duodenalis* [25]. Antibody-based assays, although sensitive, cannot distinguish previous exposure from active infection and can cross-react with other helminths, posing challenges in field conditions [2]. Nonetheless, such assays are vital for diagnosing travellers, migrants, and occasionally exposed individuals [26]. In children, the infection is typically recent, simplifying the interpretation of positive serology. Although cross-reactions can lead to potential overtreatment, underdiagnosis could result in severe long-term complications. Screening strategies for patients from endemic areas remain crucial to prevent severe sequelae, and the possibility of community transmission should be considered, since with climate change and globalisation, some parasitosis might become endemic. In fact, cases of autochthonous schistosomiasis have recently been reported in Europe [18, 27], highlighting the need for maintaining adequate epidemiological surveillance. Standardisation of diagnostic methods with a dual approach, combining serology and direct methods, is emphasised [28].

Few series include longitudinal follow-up, and therefore the effect of treatment on absolute eosinophil count, serological optical density, or IgE remains poorly understood. A notable feature of the UCP-LF CAA test is its ability to detect a decrease in antigen levels shortly after praziquantel treatment, enabling effective individual treatment monitoring [10]. Efforts are ongoing to develop a user-friendly antigen detection test using finger prick blood (CAA-RDT), but these tests are not yet available for routine clinical use [29]. Regarding antibody detection methods, different studies have shown an increase in anti-parasite IgM and IgE titres 6 weeks after treatment with praziquantel, which could play a protective role against reinfection in children [30]. Studies including adults have shown how most serological assays yield positive results for at least 2 years after treatment and in many cases even longer [31]. Therefore, most guidelines do not recommend serological follow-up. However, our work suggests that longitudinal optical density over time could be helpful for monitoring the response to treatment in certain parasitosis, and sometimes is needed to discard cross-reaction. A progressive decline in absolute eosinophil counts, IgE titres, and serological optical density was noted within the first 6 months after treatment, further accentuated after 12 months.

With the WHO’s ambitious goal of a “world free of schistosomiasis,” advocating for empiric treatment in school-age children living in endemic areas, achieving 75% coverage is targeted to eliminate the disease by 2025. However, in non-endemic countries with available serological testing, we argue for screening, given that the absence of symptoms or abnormal laboratory findings cannot rule out the infection. Schistosomiasis remains a frequently imported parasitosis, underscoring the importance of ongoing vigilance [9, 32].

Abdominal ultrasound abnormalities were observed in 6 of 13 patients, all diagnosed with urinary schistosomiasis by the direct method. Standardisation of diagnostic methods with a dual approach, combining serology and direct methods, is emphasised [28].

Moreover, the lack of paediatric praziquantel formulations for preschool children in many areas and the challenge of adherence due to the bitter taste of existing tablets necessitates caution in treatment [33, 34]. Pharmacokinetics data are typically scarce in the paediatric population, and existing data suggest that higher doses (> 60 mg/Kg) are needed [35]. Therefore, treatment warrants additional caution. Praziquantel was well tolerated in our series, with a median dose of 40 mg/kg, which is below the recommended dosage for children according to evidence. The Pediatric Praziquantel Consortium has produced a paediatric tablet meeting a previously suggested target product profile [36]. This formulation—a smaller, orally dispersible tablet with a masked taste—has successfully undergone phase II clinical trials, and phase III trials are currently underway, and a positive opinion is expected by the EMA by the end of 2023 [37].

The study's main limitations include its retrospective, single-centre design, small sample size, the absence of long-term follow-up data for many patients, and the fact that not all determinations were available at all time-points. Despite the standardised protocol, complementary tests were performed according to the treating clinician, and not all patients were screened for other helminths or underwent abdominal ultrasounds. Most of the commercial kits are based on *S. mansoni* antigens and are therefore less sensitive to detect other species [3]. Despite these limitations, this study represents one of the largest series of paediatric patients in a non-endemic country, including longitudinal follow-up with serological results.

The burden of disease related to schistosomiasis is significant. Given the frequently asymptomatic nature of the infection and the unreliability of eosinophilia as a screening tool, we strongly advocate for screening migrant and internationally adopted children. Serological tests for schistosomiasis should be offered to children arriving in Europe from endemic areas (particularly those from Sub-Saharan Africa and Asia), as treatment can effectively prevent long-term sequelae. Absolute eosinophil count, total IgE, and ELISA optical density can be useful at diagnosis and during follow-up in combination with morbidity questionnaires, although new techniques are very much needed to monitor treatment response.

## Data Availability

No datasets were generated or analysed during the current study.
